# Opportunities for CAR-T Cell Immunotherapy in HIV Cure

**DOI:** 10.3390/v15030789

**Published:** 2023-03-19

**Authors:** Gerard Campos-Gonzalez, Javier Martinez-Picado, Talia Velasco-Hernandez, Maria Salgado

**Affiliations:** 1 IrsiCaixa AIDS Research Institute, 08916 Badalona, Spain; 2 University of Vic—Central University of Catalonia (UVic-UCC), 08500 Vic, Spain; 3 Catalan Institution for Research and Advanced Studies (ICREA), 08010 Barcelona, Spain; 4 CIBER de Enfermedades Infecciosas, Instituto de Salud Carlos III, 28029 Madrid, Spain; 5 Germans Trias i Pujol Research Institute (IGTP), 08916 Badalona, Spain; 6 Josep Carreras Leukaemia Research Institute, 08036 Barcelona, Spain; 7 RICORS-TERAV, ISCIII, 28029 Madrid, Spain

**Keywords:** CAR-T cells, chimeric antigen receptor, immunotherapy, HIV, HIV reservoir, latency reversal agents

## Abstract

Chimeric antigen receptor (CAR) technology is having a huge impact in the blood malignancy field and is becoming a well-established therapy for many types of leukaemia. In recent decades, efforts have been made to demonstrate that CAR-T cells have potential as a therapy to achieve a sterilizing cure for human immunodeficiency virus (HIV) infection. However, translation of this technology to the HIV scenario has not been easy, as many challenges have appeared along the way that hinder the consolidation of CAR-T cells as a putative therapy. Here, we review the origin and development of CAR-T cells, describe the advantages of CAR-T cell therapy in comparison with other therapies, and describe the major obstacles currently faced regarding application of this technology in the HIV field, specifically, viral escape, CAR-T cell infectivity, and accessibility to hidden reservoirs. Nonetheless, promising results in successfully tackling some of these issues that have been obtained in clinical trials suggest a bright future for CAR-T cells as a consolidated therapy.

## 1. Introduction

A landmark in the history of human immunodeficiency virus (HIV) infection was long-term suppression of the viral replication, achieved in 1996 by the administration of triple-drug therapy, namely, combination antiretroviral therapy (cART) [1]. This breakthrough made a cure for HIV seem possible for the first time. However, almost simultaneously, it was demonstrated that CD4^+^ T cells from people with HIV contained integrated HIV-DNA [2]. This phenomenon, which came to be known as latent infection, is the main reason why antiretroviral therapy is not capable of completely eliminating HIV infection [3]. Although the reservoir in blood decreases during cART treatment, after a first reduction, a relatively stable pool of latently infected cells remains [4,5,6,7]. Currently, the persistence of the HIV-1 reservoir is considered a major barrier to achieving virus eradication, even though there is some evidence that curing HIV-1 is a feasible goal.

A small group of people with HIV, known as exceptional elite controllers, are living proof that cure is possible, even if only a functional cure. Exceptional elite controllers have lived with no viral replication, in some cases for more than 30 years [8], and in some cases, presenting immune seroreversion [9]. The causes of this good control are multifactorial, but in most cases, elite controllers have strong immune cytotoxic T lymphocyte (CTL)-mediated responses, and this, in combination with very low viral reservoir levels, leads to long-term control of viral replication and pathogenesis in the absence of cART [10,11]. Other cohorts of post-treatment controllers [12] and individuals that naturally reduce HIV reservoirs during cART [13,14] are being also studied to confirm that HIV reservoirs can be blocked and reduced. All this evidence suggests that functional cure, even if infrequent, is feasible.

Much more complicated is achieving a fully sterilizing cure. So far, only three cases of HIV cure through a medical intervention have been reported in the scientific literature: the Berlin, London, and Düsseldorf patients [15,16,17,18]. Their cure resulted from an allogeneic hematopoietic stem cell transplant (allo-HSCT) with a homozygous donor for *CCR5Δ32* deletion to prevent HIV-1 infection of the graft. Additional studies of transplanted HIV individuals have demonstrated that the alloreactivity related to a stem cell transplant is sufficient to achieve a reduction in HIV reservoirs, and frequently causes a delay in viral rebound after cART treatment interruption, even when the protective effect of *CCR5Δ32* deletion of the donor is lacking [19,20]. To achieve the goal of a sterilizing cure, therefore, an approach is needed that can mimic the alloreactivity that reduces the HIV reservoir but is implemented in a context outside of a high-risk procedure such as the allo-HSCT.

Different strategies have been tested to enhance immune responses that are capable of killing HIV infected cells. Certain therapeutic vaccination strategies have achieved interesting results in increasing HIV-1 specific immune responses, but have a limited impact on the reduction of HIV reservoirs [21]. Additionally, inhibitory receptors, also called immune checkpoint inhibitors, can exert their influence on T-cell responses, although to date results have been marginal [22]. Chimeric antigen receptor (CAR) T cell therapy, in contrast, is postulated as the most suitable strategy to enhance T cell responses to levels that can mimic allo-HSCT reactivity. Good model design for CAR-T cells with tropism for cells infected with HIV has been undergoing development for several years. In this review, we compile the main contributions to the field and describe some of the challenges faced in regard to achieving a sterilizing cure with HIV-specific CAR-T cell therapy.

## 2. Development of the CAR Technology

### 2.1. Early Studies of CAR-T Cells

The chimeric antigen receptor (CAR) T cell technology was first conceptualized in the 1980s by Eshhar and Gross [23] with the idea of directing T cell responses through genetic editing of T cell receptors (TCRs). The earliest clinical application for this new tool was as a putative therapy for HIV infection in which cytolytic CD8^+^ T cells (CTLs) were genetically modified to express natural HIV ligand CD4 as an extracellular domain, along with a transmembrane domain and a signal transducing intracellular domain CD3ζ [24]. Thus, on HIV ligand recognition by the CD4 extracellular domain, a CAR-T cell cytotoxic response would be generated against cells expressing HIV antigen gp120. This response would be target-specific and also major histocompatibility complex (MHC)-independent, meaning that HIV’s ability to downregulate surface expression of MHC I to produce immune escape from CTLs would not affect CAR-T cells [23,25,26]. Testing in the early 1990s in in vitro assays showed that CAR-T cells were able to specifically recognize and lyse cells expressing HIV gp120 [26,27]. Based on in vitro promising results, in vivo assays and clinical trials were performed, and although the therapy was shown to be safe and feasible and to present optimal cell survival, there was no control of the infection or of virus relapse (Table 1) [24,28,29].

### 2.2. New Generations of CARs-T Cells

Several factors could explain the poor effect observed in the early experiments, such as inefficient ex vivo treatment of CD8^+^ T cells [30]. Regarding CAR design, new alternatives gradually emerged to improve the first generation of CAR-T cells. The second generation of CAR-T cells added intracellular co-stimulatory domains such as CD28 or 4-1BB to the CAR construct. These domains would be linked to the intracellular signal transducing domain CD3ζ, contributing to lymphocyte activation, proliferation, sustained response, and prolonged life of the CAR-T cells [32,33]. While the CD28 costimulatory domain promoted higher cytokine production and better control of virus replication in vitro, CAR-T cells containing 4-1BB costimulatory domain performed better at controlling HIV infection in vivo [34]. Third and fourth generations of CAR-T cells subsequently appeared. Third-generation CARs combined different costimulatory domains to generate a stronger response by augmenting cytokine production and, therefore, their killing ability [35]. Fourth-generation CAR-T cells, or T cells redirected for universal cytokine-mediated killing (TRUCK) included an intracellular interleukin (IL)-12 domain in the corresponding CAR construct, so that CAR expression would be accompanied by the release of transgenic cytokine-like IL-12 upon CAR activation; this would, in turn, enhance activation of T cells and attract other innate immune cells [36]. A fifth generation of CAR-T cells is currently being explored, based on second-generation design. This new proposal adds a truncated domain of IL2Rβ and a STAT3-binding motif to the CD28 and CD3ζ intracellular domains, resulting in enhanced proliferation in vitro and prolonged persistence in vivo [37].

### 2.3. Translation of the CAR-T Cell Technology to the Clinic

Clinical trials using second-generation CAR-T cells obtained better results than the earlier design. Promising results were presented in the field of cancer, specifically in haematological malignancies, targeting ligands such as CD19, expressed in B cells [38,39,40,41]. This led to approval by the Food and Drug Administration (FDA) of tisangenlecleucel (Kymriah^TM^) in August 2017 as the first CAR-T cell therapy, aimed at treating B cell acute lymphoblastic leukaemia (ALL) in paediatric and young adult patients and also diffuse large B cell lymphoma in adult patients [42]. Another example of CAR-T cell therapy is axicabtagene ciloleucel (Yescarta^TM^), approved by the FDA in October 2017; this therapy was also aimed at treating several subtypes of B cell lymphoma with a second-generation CAR designed to target all CD19-expressing cells [42]. FDA approval for Kymriah^TM^ and Yescarta^TM^ were followed by others, also in the haematological malignancies field, in subsequent years [43].

However, for HIV infection, results in clinical trials testing CAR-T cells were not as promising (Table 1). In 2000, Mitsuyasu and colleagues [28] genetically engineered first-generation CD4-based CAR-T cells that targeted the HIV envelope protein gp120. The treatment consisted of administering a single dose of autologous CD4-based CAR-T cells with or without exogenous IL-12 to 24 people with HIV. It was found, in the treated individuals in comparison with non-treated individuals, that although there was a significant decrease in HIV RNA associated with rectal tissue, no significant mean change in plasma HIV RNA occurred, or blood proviral DNA was detected. Similarly, in a phase-II randomized study, Deeks and colleagues [29] also observed an absence of significant differences in viral reservoirs between study groups following cART with CD4-based CAR-T cells administered to 40 people with HIV. Nevertheless, both studies reported their satisfaction with the safety and feasibility of the therapy. They also concluded that sustained survival of CAR-T cells in circulation was achieved after optimal ex vivo treatment, a finding that was subsequently corroborated elsewhere [24,30].

## 3. Difficulties and New Strategies to Achieve HIV Cure with CAR-T Cells

CAR-T cell therapies have been applied in many clinical assays for haematological malignancies, with excellent results [44] that demonstrate the safety, feasibility, efficacy, and durable effect of the therapy. Based on these promising outcomes, the HIV field has begun to view CAR-T cell therapy as a potentially promising HIV cure strategy. Unlike other HIV therapeutic approaches, CAR-T cell treatment would be a targeted therapy that would aim to achieve a sterilizing HIV cure, thereby addressing one of the major drawbacks to finding a cure, i.e., completely eliminating the HIV reservoir from the organism [45]. The objective would be for the CAR-T cells to proliferate and engraft to a degree that would ensure a long-lasting therapeutic effect, while avoiding the side effect of graph-*versus*-host disease (GvHD), given that T cells are autologous. In this way, the therapy would generate immunogenic memory and surveillance directed at fully eliminating HIV-infected cells while avoiding any chance of viral rebound. Although fewer clinical studies have been performed with HIV, the authors are all satisfied that the therapy has been demonstrated to be safe and feasible [28,29], which is a key starting point.

However, the blood cancer and HIV scenarios are very different. Blood malignancies present higher amounts of the targeted antigen with constant regions, and this makes it easier and more accessible for the CAR-T cells to elicit their cytotoxic response. The variability of the gp120 protein and the fact that gp120 expressing cells do not represent the total of HIV infected cells may compromise the efficacy of the CAR-T cells in achieving a putative sterilizing cure. Additionally, the fact that the first CAR-T cells were designed with the CD4 motif raised the possibility that new viral particles could infect the engineered cells. To bypass these three important caveats, different approaches have been developed within the HIV field.

### 3.1. Avoiding CAR-T Cell Infection

**Figure 1:**
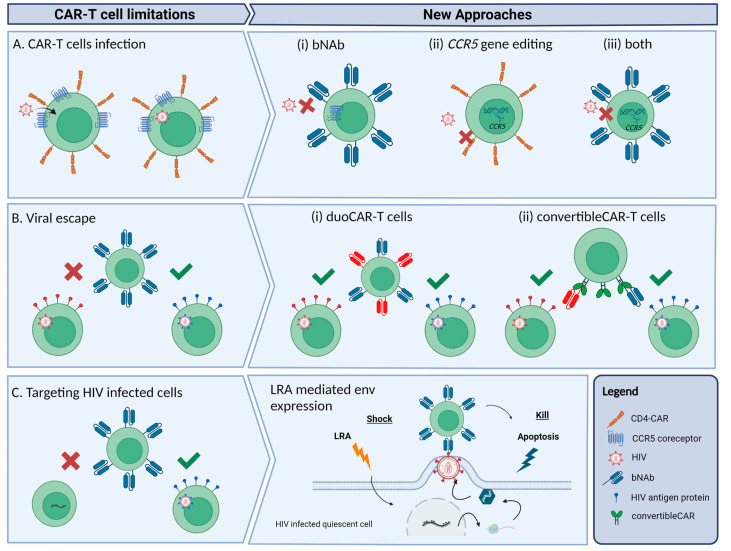
Schematic representation of the main limitations of CAR-T cells as a therapy for HIV and new approaches to overcome the limitations. (**A**) CD4-CAR-T cells can be infected by HIV-1 through interaction with CD4 and CCR5 receptors present in the CAR. Alternative approaches proposed: (i) use of bNAbs to build the CAR; (ii) deletion of the *CCR5* gene through gene editing techniques to prevent infection; or (iii) both approaches combined. (**B**) The high mutation rate of HIV-1 can generate HIV-1 strains resistant to the CAR-T cell cytotoxic effect. Some of the new CAR-T cell designs: (i) duoCAR-T cells, which are able to target more than one gp160 epitope, and (ii) convertibleCAR-T built with a common binding site for a variety of gp160-specific bNAbs that can mitigate viral escape. (**C**) Latently infected cells from reservoirs are not accessible to CAR-T cells, but shock-and-kill approaches such as using LRAs can transcriptionally reactivate these cells making them accessible targets for CAR-T cells.

A major disadvantage of CD4-based CAR-T cells is their susceptibility to infection by HIV, given that CD4 is the natural ligand for the gp120 envelope protein of HIV [46,47]. To address this drawback, in place of CD4 as the extracellular antigen recognition domain, broadly neutralizing antibodies (bNAbs) against HIV are engineered in the CAR construct. Approximately 1–2% of HIV-infected individuals generate these antibodies [48], which present a high affinity for the HIV envelope protein. In recent years, many bNAbs have been isolated that target different epitopes of the gp160 protein [49]. Hale and colleagues [50] predicted that the inclusion of single-chain variable fragments (scFvs) of these antibodies as the antigen recognition domain of the CAR construct would generate potent anti-HIV activity in CAR-T cells, and demonstrated that bNAbs are compatible with CAR technology by producing four different bNAb-based CAR-T cells with robust anti-HIV activity in vitro [50]. Overall, in comparison with CD4-based CAR-T cell designs, monoclonal antibody-based CAR demonstrated a higher neutralizing capacity for different HIV-1 strains and showed an ability to avoid HIV infection (Figure 1Ai). Complementarily, Liu and colleagues connected the scFvs of a bNAb (VRC01) to a third-generation CAR moiety. It was demonstrated that these CAR-T cells could effectively eliminate the HIV-reactivated infected CD4^+^ T lymphocytes, i.e., the quiescent cells from the reservoir harbouring the latent provirus [31]. Several clinical trials are currently ongoing, in which second- and third-generation bNAb-based CAR-T cells are being tested in HIV-infected individuals (Table 1). One recently published clinical trial by Liu and colleagues [31], based on in vitro testing, showed promising results for the safety and effectiveness of bNAb-based CAR-T cell therapy targeting the HIV reservoir in individuals receiving cART. In addition to the safety of the therapy and the long-term in vivo persistence of the CAR-T cells, the clinical trial demonstrated delayed viral rebound when cART was discontinued and also accelerated depletion of the HIV reservoir during cART. However, viral escape from CAR-T cells due to spontaneous mutations generated resistant variants that led to viral rebound [31].

Prevention of CAR-T cell infection is also key to maintaining CAR-T cell persistence and anti-HIV activity in vivo. One approach to avoiding infection of CAR-T cells is by knocking out the *CCR5* gene via ex vivo genetic editing using zinc-finger nucleases (ZFNs), transcription activator-like nucleases (TALENs) or CRISPR-Cas9 technology [34,51]. The *CCR5* gene encodes for the main coreceptor for HIV entry and infection, so knockout of this gene in CAR-T cells may help generate permanent resistance to HIV infection. Indeed, it has been demonstrated that individuals with a non-functional allelic variant of this gene (*CCR5Δ32*) are protected from CCR5-tropic HIV infection [52]. Moreover, the three published cases of cured individuals known to date, i.e., the Berlin, London, and Dusseldorf patients [16,18,53,54], underwent allo-HSCT with cells from donors who were homozygous for the *CCR5* deletion (*CCR5Δ32/Δ32*). In a clinical trial, Tebas and colleagues [55] demonstrated the safety and feasibility of a therapy that attempted to mimic the genetic inherited resistance to HIV in individuals carrying the *CCR5Δ32/Δ32* deletion. Autologous CD4^+^ T cells were treated ex vivo to knock out *CCR5* using ZFNs and were then reinfused in individuals that had been on cART. It was demonstrated that the survival rate was higher in the modified CD4^+^ T cell group than in the unmodified CD4^+^ T cell group, suggesting that *CCR5* knockout confers a selective advantage on CD4^+^ T cells in HIV-infected individuals [55]. In a more recent clinical trial by the same group, it was observed that the HIV-specific immune response was improved in 14 people with HIV infused with *CCR5* gene-edited CD4^+^ T cells and also that some of the participants controlled the HIV rebound after treatment interruption [56]. The same authors also described a case of viral escape, indicating the potential of HIV-resistant CD4^+^ T cells to reinvigorate an HIV-specific immune response [56].

In an in vivo assay using nonhuman primates, CD4^+^ CAR-T cells were genetically modified to knock out the *CCR5* gene in order to protect the CAR-T cells from simian HIV (SHIV) infection [57] (Figure 1Aii). One of the main studies tested the feasibility of a therapy combining bNAb-based second-generation CAR-T cells homozygous for the *CCR5Δ32* deletion. Used was homology-directed repair (HDR) to specifically insert bNAb-based CAR in the *CCR5* locus, thereby disrupting the *CCR5* gene while simultaneously introducing the CAR construct (Figure 1Aiii). It was observed that the *CCR5*-modified bNAb-based CAR-T cells performed better in maintaining control of viral replication than the bNAb-based CAR-T cells without the *CCR5Δ32* deletion. Those results support the idea that, in the presence of active HIV replication, *CCR5* disruption is critical to avoiding the infection of effector cells, and thus to maintaining anti-HIV activity [50]. A clinical trial is currently ongoing using this strategy of CAR-T cells modified with *CCR5* ablation (Table 1).

Overall, because CD4 and CCR5 are HIV primary coreceptors, the use of CD4^+^ T cells for the CAR technology may enhance the risk of infection of CAR-T cells. However, it has been clearly proven that *CCR5Δ32* deletion confers a selective advantage on CD4^+^ T cells in individuals with HIV [52]. Nonetheless, the use of CD8^+^ T cells for the CAR-T cell technology might be a better approach, not only because HIV-specific CD8^+^ T cells play a key role in active control of viremia, but also because CAR-T cell infection will be avoided. Moreover, some studies have shown that HIV-specific CD4^+^ T cells actively collaborate with HIV-specific CD8^+^ T cells in enhancing in vivo persistence and survival of the latter. That they do this by upregulating certain cytokines, such as IL-2 or IL-21 [58,59], would suggest that the perfect CAR-T cell therapy would have to be a combination of both cell types to achieve the desired synergistic antiviral effect.

### 3.2. Reducing Viral Escape

A drawback to the CAR-T cell approach is the immune escape that the viral protein gp120 can render. HIV can spontaneously mutate its envelope protein and produce immune escape from CAR-T cells, thereby favouring rebound of resistant viruses [60,61]. In their clinical trial, Liu and colleagues [31] observed that the rebounded virus strains were resistant to CD8^+^ CAR-T cell-mediated cytotoxicity, which confirms the effectiveness of CAR-T cell therapy in restricting viral replication and forcing mutations in viruses to generate immune escape. The same authors also noted that viraemia rebounded sooner in trial participants with fewer CD4^+^ T cells but took longer in trial participants with a higher number of CD4^+^ T cells. Although no significant data regarding this issue was reported for the clinical trial, the results would suggest that a possible collaboration between CD8^+^ CAR-T cells and CD4^+^ T cells might lead to a delay in viral rebound [31]. A possible solution for viral escape is the dual or duoCAR-T cells, which target multiple highly conserved sites in the HIV-1 envelope glycoprotein gp160 [62]. Instead of aiming for just one epitope of the HIV envelope protein, CARs are designed to express two or even three gp120/gp41 domains per construct, thereby specifically recognizing and effectively killing HIV infected cells (Figure 1Bi). In comparison with single-site targeting CAR-T cells, immune escape due to HIV genetic diversity is thus delayed or even avoided [62,63]. A recent study showed that multiple-site targeting CAR-T cells consistently demonstrated greater suppressive activity against HIV-1 in vitro and in vivo than single-site targeting CAR-T cells, and also managed to control the HIV reservoir size by eliminating HIV-infected cells as they arose [62]. A clinical phase I/IIa study by the same group is currently recruiting participants to assess the safety and viability of duoCAR-T cell therapy on cART-treated HIV-infected individuals (Table 1). Another similar approach is the convertibleCAR-T cell technology, which also targets multiple gp160 epitopes through a platform where bNAbs can be coupled with a mutated ligand that specifically binds to the CAR (Figure 1Bii) [64]. Initial in vitro results with this technology showed that, within 48 h, convertibleCAR-T cells killed more than half of the inducible reservoir found in blood, what makes it a promising tool for attacking the latent HIV reservoir.

### 3.3. Targeting HIV Infected Cells

As we have previously discussed, a key factor makes CAR-T cell therapies more successful in the blood cancer scenario. Blood malignancies are characterized by uncontrolled multiplication of the target cell, which translates into an enormous amount of antigen, and this makes the cell more easily accessible for the CAR-T cells to elicit a cytotoxic response. In contrast, the fact that the amount of antigen in people with HIV on cART is lower requires a more greatly target-specific approach to CAR-T cell therapy. In a context of suppressive cART therapy, HIV-infected cells are mostly transcriptionally silent, and this translates into minimal amounts of available targets for CAR-T cells.

One approach that could help to activate reservoir cells and enhance target availability is the shock-and-kill strategy, which aims to reactivate the latent provirus through latency reversal agents (LRAs). With the LRAs, latent provirus should become transcriptionally active and cells should therefore express viral antigens in their membranes, facilitating virus elimination by anti-HIV effector cells [65] (Figure 1C). Liu and colleagues [66] reactivated latently infected CD4^+^ T cells using several LRAs and then cocultured the activated T cells with bNAb-based third-generation-CAR-T cells. They observed a significant decrease in viral RNA in cocultures with CD8^+^ CAR-T cells; in contrast, cocultures with CD8^+^ T cells from the same subjects showed a minimal reduction in viral RNA. Moreover, this antiviral activity in cocultures with CD8^+^ CAR-T cells was maintained over time, suggesting that CD8^+^ CAR-T cells are capable of recognizing and neutralizing reactivated HIV-infected CD4^+^ T cells in vitro [66].

Unfortunately, clinical trials deploying the shock-and-kill strategy have concluded that, even after LRA administration, there was a poor cytotoxic anti-HIV response from host CTLs, which led to a failure to fully eliminate reservoir cells due to immune exhaustion [67]. Another factor contributing to the poor cytotoxic anti-HIV response is the fact that some LRAs have been shown to present an inhibitory effect on cytolytic CD8^+^ T cells [68]. This is linked to the fact that, as suggested by recent studies, HIV reservoir levels in chronically infected individuals are underestimated and may, in fact, be up to 60 times higher than previously estimated [69]. Thus, the non-induced intact proviruses, being replication-competent, would maintain the reservoir viral load and prevent complete elimination and a sterilizing cure, given that the non-induced proviruses may be reactivated later on [69]. These results imply that there is a need for more potent LRAs that could fully induce all latent proviruses so that CAR-T cells can access the entire reservoir formed by the non-induced intact proviruses and exert their kill strategy to fully eliminate the reservoir.

Given that the most important barrier is revealing the latently HIV-infected cells to the immune effector cells so that these can elicit an effective antiviral response, another strategy that has been proposed is to physically expose those cells rather than try to reactivate them. Latently infected cells from the reservoir are mostly resting memory CD4^+^ T cells, presenting a low proliferation rate, mainly by homeostatic proliferation through IL-7 [70], and found in several anatomical locations such as the lymph nodes, gut-associated lymph tissue (GALT) [53], genital tract [71], and brain [72]. Some of these locations, specifically the brain and the lymph node B cell germinal centres, are sanctuaries, meaning that the cells inside are protected from cART or CTL penetration [73], thereby hindering the complete eradication of the reservoir. Studies of HIV-infected patients that have received allo-HSCT have found traces of HIV-specific T cell responses, despite proviral HIV DNA being undetectable (even to the most sensitive techniques), indicating that infected cells can persist in inaccessible sanctuaries [20,74]. A recent study has proposed a novel strategy aimed at using the CAR-T cell technology to access these sanctuaries and force exposure of the latent HIV-infected cells to the effector anti-HIV cells. Peterson and colleagues [75] designed CAR-T cells targeting CD20^+^ expressing cells with the idea of disrupting lymph node B cell germinal centres and exposing latently infected T cells in sanctuaries to effector CTLs, thus enhancing the cytotoxic response. They observed that, although the antiCD20-CAR-T cells effectively travelled to and disrupted the B cell germinal centres, there was no overall significant response regarding reduction of viral DNA or RNA in the analysed tissues. Another study attempted to eliminate the HIV reservoir in follicular dendritic cells (FDCs) using an HIV-specific CAR construct that targeted the HIV envelope protein by enabling expression of CD4 domains 1 and 2 and expression of the carbohydrate recognition domain of mannose-binding lectin (MBL) [76]. Although their CAR-T cells proved to specifically recognize and lyse CD4^+^ T cells expressing the HIV glycoprotein, they did not specifically lyse HIV-infected FDCs, and overall, their CAR-T cell therapy design was unable to reduce the FDC reservoir [76]. Hence, it is important to both ensure efficient transport of the CAR-T cells to the tissue sanctuaries and to maintain their potent antiviral effect once the effector cells make contact with the reservoir.

## 4. Conclusions

CAR-T cell therapy is a well-established technology that is having a crucial impact in the field of blood cancer. A similar approach is being explored in the field of HIV, with some promising results in clinical trials using new generations of CAR-T cells and combining different strategies, including shock-and-kill. However, a number of important obstacles still need to be addressed, namely, avoiding CAR-T cell infection from the virus, reducing viral escape, and achieving complete elimination of HIV reservoirs that are transcriptionally silent. New studies are generating promising and original approaches, including the duoCAR strategy, with encouraging results, and it may be that a combination of new approaches will be crucial to overcoming the above-mentioned obstacles. Although the number of clinical trials testing CAR-T cells for HIV are still few, they have all demonstrated that the therapy is safe and feasible, which is a key starting point for finding a cure for HIV.

## Figures and Tables

**Table 1:** Overview of CAR-T cell clinical trials in HIV field.

Trial Registration	Study Title	Start Date	Phase	Country	CAR Generation	Type of CAR	Outcome	Reference
**-**	Prolonged survival and tissue trafficking following adoptive transfer of CD4ζ gene-modified autologous CD4^+^ and CD8^+^ T cells in human immunodeficiency virus–infected subjects	1999 *	Phase II	USA	First	CD4ζ-based	Validation of the feasibility and antiviral activity	Mitsuyasu et al. *Blood* **2000**. [28]
**-**	Long-term in vivo survival of receptor-modified syngeneic T cells in patients with human immunodeficiency virus infection	1999 *	Phase I	USA	First	CD4ζ-based	Prove that administration is safe	Walker et al. *Blood* **2000**. [24]
**-**	A phase II randomized study of HIV-specific T-cell gene therapy in subjects with undetectable plasma viremia on combination antiretroviral therapy	2002 *	Phase II Randomized	USA	First	CD4ζ-based	Confirmed safety and feasibility, but no effect on HIV reservoirs	Deeks et al. *Mol. Ther*. **2002**. [29]
**NCT01013415**	A phase I/II study of the safety, survival, and trafficking of autologous CD4-ζ gene-modified T cells with and without extension Interleukin-2 in HIV infected patients	2001	Phase I Non-Randomized	USA	First	CD4ζ-based	Safety and long term persistence of modified T cells	Scholler et al. *Sci. Trasl. Med*. **2012**. [30]
**NCT03240328**	The effect of CAR-T cell therapy on the reconstitution of HIV-specific immune function	2017	Phase I	China	Third	bNAb-based	Long term in vivo persistence and no safety concerns	Liu et al. *J. Clin. Invest*. **2021**. [31]
**NCT03617198**	A pilot study of T cells genetically modified by Zinc Finger Nucleases SB-728mR and CD4 chimeric antigen receptor in HIV-infected subjects	2019	Phase I Randomized	USA	Second	*CCR5* ZFN-treated CD4^+^	Ongoing	-
**NCT04648046**	Safety and anti-HIV activity of autologous CD4^+^ and CD8^+^ T cells transduced with a lentiviral vector encoding bi-specific anti-gp120 CAR molecules (LVgp120duoCAR-T) in anti-retroviral drug-treated HIV-1 infection	2021	Phase I/IIa Non-Randomized	USA	Second	CD4-based duoCAR	Ongoing	-

* Submission date of publication because study start date is not available.

## Data Availability

Not applicable.
